# Genome Sequences of *Cyberlindnera fabianii* 65, *Pichia kudriavzevii* 129, and *Saccharomyces cerevisiae* 131 Isolated from Fermented Masau Fruits in Zimbabwe

**DOI:** 10.1128/genomeA.00064-17

**Published:** 2017-04-06

**Authors:** Irma M. H. van Rijswijck, Martijn F. L. Derks, Tjakko Abee, Dick de Ridder, Eddy J. Smid

**Affiliations:** a Laboratory of Food Microbiology, Wageningen University & Research, Wageningen Campus, Wageningen, The Netherlands; b Bioinformatics, Wageningen University & Research, Wageningen Campus, Wageningen, The Netherlands; c Animal Breeding and Genomics Centre, Wageningen University & Research, Wageningen Campus, Wageningen, The Netherlands

## Abstract

*Cyberlindnera fabianii* 65, *Pichia kudriavzevii* 129, and *Saccharomyces cerevisiae* 131 have been isolated from the microbiota of fermented masau fruits. *C. fabianii* and *P. kudriavzevii* especially harbor promising features for biotechnology and food applications. Here, we present the draft annotated genome sequences of these isolates.

## GENOME ANNOUNCEMENT

*Cyberlindnera fabianii* 65 [previously known as *Lindnera fabianii*, *Hansenula fabianii*, and *Pichia fabianii* (1, 2)], *Pichia kudriavzevii* 129 [previously known as *Issatchenkia orientalis* (2)], and *Saccharomyces cerevisiae* 131 have been isolated from the microbiota of fermented masau fruits (*Ziziphus mauritiana*) in Zimbabwe (3, 4).

All three species are found regularly in (fermented) food products (3, 5–14) but also occasionally in clinical sources (15–17). Nevertheless, *P. kudriavzevii* and *S. cerevisiae* were given the status of generally recognized as safe by the Food and Drug Administration (FDA) (18). *C. fabianii* 65 and *P. kudriavzevii* 129 especially harbor promising features for food fermentation applications, such as the production of extended aroma profiles (19). To further explore these features, the draft genomes of these wild isolates were investigated and annotated.

DNA was sequenced using Illumina MiSeq paired-end (2 × 251 bp) sequencing technology, with total depths of coverage of 83.3× (*C. fabianii* 65), 83.3× (*P. kudriavzevii* 129), and 100× (*S. cerevisiae* 131) based on a 12-Mb genome size. Moreover, PacBio sequencing was performed with total depths of coverage of 36.6× (*C. fabianii* 65), 32.1× (*P. kudriavzevii* 129), and 22.2× (*S. cerevisiae* 131). We performed hybrid assemblies using DBG2OLC with Illumina and PacBio data (20). PBJelly (21) was used for further scaffolding. The final assemblies were polished with Sparc (PacBio) (22) and Pilon (Illumina) (23). MAKER2 (24) was used to annotate the genomes using protein homology evidence from all available fungi in the Swiss-Prot database (25). *De novo* gene predictors Augustus (26) and SNAP (27) were trained using *Pichia stipitis* genome sequence data for *C. fabianii* 65 and *P. kudriavzevii* 129 and *Saccharomyces cerevisiae* for *S. cerevisiae* 131. Functional annotation was performed using BLASTp (28) against Swiss-Prot (25). Protein domains and gene ontology terms were assigned using InterProScan (29). BUSCO (30) analysis showed that more than 90% of the core fungal genes are present in all three assemblies (Table 1). The G+C percentage of *C. fabianii* 65 is 44.4% but is lower for *P. kudriavzevii* 129 (38.5%) and *S. cerevisiae* 131 (38.1%). Other assembly and annotation statistics are listed in Table 1.

**TABLE 1:** Assembly characteristics of three fungal genome sequences

Species	*N*_50_ length (bp) (index)	No. of sequences	Assembly size (bp)	No. of genes	Accession no.	BUSCO gene completeness (%)
*C. fabianii* 65	1,227,680 (4)	25	12,188,250	5,509	MPUK00000000	91.7
*P. kudriavzevii* 129	336,598 (11)	260	11,679,144	5,470	MQVM00000000	90.6
*S. cerevisiae* 131	111,157 (34)	236	12,005,589	5,445	MQVN00000000	91.7

The genome sequences and gene annotations can now be used to develop novel molecular tools to unravel the full metabolic repertoire of the two nonconventional yeasts compared to *S. cerevisiae* 131. Additionally, links between phenotypes and genotypes, as well as comparative genomic studies among the three species, will reveal opportunities for industrial applications of *C. fabianii* 65 and *P. kudriavzevii* 129.

### Accession number(s).

The annotated genome sequences are deposited at DDBJ/EMBL/Genbank under the accession numbers listed in Table 1.
